# Diseases That Kill the Most People

**Published:** 1886-02

**Authors:** 


### DISEASES THAT KILL THE MOST PEOPLE,

"It is a fact wel-known to the medical profession," said a prominent physician the other day, "that much less harm is done in New York by those diseases most feared by the public and the Health Board than by the diseases which cause little alarm in the community. The so-called plagues are not likely to cause much havoc in this latitude, particularly in a city where the sanitary regulations are good. No scourge is so much feared as Asiatic cholera, yet the deaths from that disease in this city during the year of the greatest epidemic were less in number than the deaths from consumption last year. Yellow fever has killed fewer people in New York in this century, than whooping Cough has destroyed in a single year. Small-pox, which is causing so much alarm at present, kills fewer persons here than does either measles, croup or whooping cough. The deaths from typhus fever in this town have been for many years less numerous than those from typhoid fever. These statements may be surprising to many residents of the city, but they can be easily substantiated by an examination of the death records which are about the only reliable records to be found in the Health Department. It would be well for the city, in my estimation, if the sanitary officers would spend more time investigating those diseases which cause the greatest mortality. It would be well, also, if some of the newspapers would call attention to the facts instead of causing useless excitement in the public mind about improbable epidemics of cholera, yellow fever or small-pox."

An examination of the death records, made by a reporter of The Tribune with the assistance of Dr. John T. Nagle, the Deputy Registrar of Vital Statistics, has brought to light some interesting contrasts between the mortality from so-called plagues in this city and the mortality from other diseases. According to the records there have been five epidemics of Asiatic cholera in New York, occurring in the years 1832, 1834, 1849, 1854 and 1866. Following is a comparison of deaths from cholera in those years with the deaths from consumption, pneumonia and bronchitis in five recent years:

| | Cholera. | | Consumption. | Pneumonia. | Bronchitis. |
|---|---|---|---|---|---|
| 1832 | 3,513 | 1880 | 4,706 | 2,822 | 1,375 |
| 1834 | 971 | 1881 | 5,312 | 3,261 | 1,511 |
| 1849 | 5,071 | 1882 | 5,247 | 3,472 | 1,583 |
| 1854 | 2,509 | 1883 | 5,290 | 3,409 | 1,435 |
| 1866 | 1,137 | 1884 | 5,235 | 3,159 | 1,485 |
| Total..... | 13,201 | Totals .. | 25,790 | 16,123 | 7,389 |

The earliest recorded epidemic of yellow fever in the city was in 1805, when 270 persons died from the disease. In 1822 there was anotber epidemics which carried off 166 persons. Since then the epidemic, have been few and not destructive, only 104 deaths having occurred from the fever. The recorded deaths from yellow fever, all told, number 540, while in each of several recent years the deaths from whooping coughs have numbered nearly 600. The deaths from small-pox have varied greatly in different years, but have averaged less than 300 a year. In 1845 the greatest epidemic of small-pox which the city had known up to that time killed 425 persons, and in 1847 the deaths from the disease numbered only 53. The worst epidemic of the disease which ever occurred in the city was in 1875, and it destroyed 1,280 lives, but in 1878 only two persons died from the smallpox. An epidemic which began in 1881 was the last serious one. Deaths in that year and in following years from several contagious diseases are given below for purposes of comparison:

| | Small Pox. | Diptheria. | Scarlet fever. | Measles. | Croup. | Whooping Cough. |
|---|---|---|---|---|---|---|
| 1881 | 451 | 2,249 | 1,964 | 429 | 1,038 | 286 |
| 1882 | 50 | 1,526 | 2,066 | 913 | 729 | 658 |
| 1883 | 12 | 1,009 | 741 | 716 | 614 | 327 |
| 1884 | 0 | 1,090 | 608 | 762 | 748 | 490 |
| Total........ | 722 | 5,873 | 5,382 | 2,820 | 3,159 | 1.761 |

There has been a gradual change in the relations of typhus fever and typhoid fever, chiefly in the prevalence of those diseases. Typhus fever continues to be the most deadly in comparison with the number of cases, but its prevalence has decreased almost in a ratio with the increase of the city's population, a fact attributed by the sanitary officials to improved regulations for the lodging houses and to the changes in travel across the ocean. The frequency of cases of typhus fever on board the old sailing vessels gave to the disease the name of "ship fever." Since the carrying of passengers across the ocean has been monoplized by the steamships, cases of the fever on vessels have been scarce. In 1850, 977 persons died in the city from typhus fever and 156 from typhoid fever. The record for 1860 was 198 deaths from typhus and 232 deaths from typhoid. In 1870 the deaths stood: 96 from typhus and 422 from typhoid. Only three persons died from typhus fever in 1880, while the deaths from typhoid fever numbered 241. There was an epidemic of typhus fever in the overcrowded lodging houses in 1881. Which carried off 160 persons, mostly vagrants, but since then the deaths from the disease have been few. The deaths from typhoid fever meanwhile have been increasing each year.

The records in the Bureau of Vital Statistics also give information which disproves a popular notion that the death rate of a city necessarily increases somewhat with the increase in population. In 1817 the death rate per 1,000 inhabitants was 20.86 and was smaller than for any previous year since 1800. The smallest recorded death rate for this city during this century was 20.79 in 1844, when the population was three times larger than in 1817. In 1877, when the population had nearly trebled again, the death rate was 24.50, which was about the average death rate for the years preceding 1844. The death rate in each of the cholera years 1832 and 1849 was greater than could be expected from the increase in the mortality due to cholera alone, but fright on account of the scourge caused many persons to die from other diseases. Below are the reported deaths in the years mentioned and in 1884, compared with the estimated population of the city in each year:

| | Deaths. | Population | Death rate per 1,000. |
|---|---|---|---|
| 1817 | 2,409 | 115,504 | 28.86 |
| 1832 | 9,975 | 224,613 | 44.41 |
| 1844 | 8,188 | 393,816 | 20.79 |
| 1849 | 22,605 | 495,266 | 45.64 |
| 1877 | 23,203 | 1,069,302 | 24.50 |
| 1884 | 35,034 | 1,356.958 | 25.82 |

